# Supplementary material for: Extent of compliance with COVID-19 prevention and control guidelines among supermarkets in Kampala Capital City and Mukono Municipality, Uganda
Source: PLoS One. 2021 Oct 28;16(10):e0258840. doi: 10.1371/journal.pone.0258840 (PMC8553093; doi:10.1371/journal.pone.0258840)
Supplement: S1 Appendix — (DOCX) [file pone.0258840.s001.docx]

# S1 Appendix. Structured Observation tool for compliance with COVID-19 prevention and control guidelines in supermarkets

**IDENTIFICATION INFORMATION**

| District | 1. Kampala 2. Mukono |
| --- | --- |
| Classification of the supermarket | 1. Large 2. Medium 3. Mini |
| Total number of staff |  |
| Total number of counter attendants at the supermarket |  |
| Does the supermarket have provisions for sale of fresh meat? | 1. Yes 2. No |
| Does the supermarket have a bakery? | 1. Yes 2. No |

| **Assessment questions** | | | | |
| --- | --- | --- | --- | --- |
| **No** | Item | Is the practice performed? | | Comments |
|  | **IPC leadership and administrative controls** |  |  |  |
|  | Are Infection Prevention and control procedures available for customers/ clients? | Yes | No |  |
|  | Are Infection Prevention and control procedures followed by customers/clients? | Yes | No |  |
|  | Does the supermarket have someone (leader) or a team in charge of infection prevention and control? | Yes | No |  |
|  | Is/are the person/s involved in enforcing customer adherence to infection prevention and control procedures active? | Yes | No |  |
|  | Have staff received job-specific training/mentorship on infection prevention and control for COVID-19? | Yes | No |  |
|  | Does anyone record clients accessing the supermarket to enable easy follow-up in case of a suspected case? | Yes | No |  |
|  | Does the team in charge of infection prevention and control have dedicated time to conduct active hand hygiene promotion? (e.g. teaching monitoring hand hygiene performance, organizing new activities) | Yes | No |  |
|  | Are there provisions for work shifts among the staff of the supermarket? | Yes | No |  |
|  | Are staffs given a leave (or offs)? | Yes | No |  |
|  | **Hand hygiene** | | | |
|  | Does the supermarket have supplies necessary for adherence to hand hygiene (e.g., soap, paper towels, sanitiser, alcohol-based hand rub, and chlorine)? | Yes | No |  |
|  | Are hand washing facilities placed at the entrance of the supermarket? | Yes | No |  |
|  | If yes, is it functional? | Yes | No |  |
|  | Do all customers wash their hands before accessing the supermarket? | Yes | No |  |
|  | If a hand washing facility is used, is it well drained? | Yes | No |  |
|  | Does the supermarket periodically monitor and record adherence to hand hygiene and provide feedback to personnel regarding their performance? | Yes | No |  |
|  | Are there posters explaining the indications for hand hygiene? | Yes | No |  |
|  | Are there posters explaining the correct use of handrub or hand sanitizer? | Yes | No |  |
|  | Are there posters explaining correct handwashing technique? | Yes | No |  |
|  | **Personal Protective Equipment (PPE)** | |  | |
|  | Does the supermarket have sufficient and appropriate PPE available and readily accessible to staff? (for example, face masks or face shields) | Yes | No |  |
|  | Is PPE correctly used by the supermarket staff? | Yes | No |  |
|  | Does the supermarket provide disposable gloves to use during the shopping? | Yes | No |  |
|  | Do all customers use the disposable gloves during their shopping? | Yes | No |  |
|  | **Temperature monitoring** |  |  |  |
|  | Does the supermarket have an infrared thermometer for screening every customer? | Yes | No |  |
|  | Does the supermarket screen every customer accessing the shopping section using an infrared thermometer (temperature gun)? | Yes | No |  |
|  | If YES, was/were screener/s maintaining distance (at least 2m) from patients? | Yes | No |  |
|  | If YES, was screener using infrared thermometer appropriately? (Thermometer held 3-5 cm from temple to get accurate reading) | Yes | No |  |
|  | If YES, is screener using flowchart? (Screener must be able to apply case definition for COVID 19) | Yes | No |  |
|  | If YES, is screener wearing appropriate PPE? (Using gloves, face shield **or** goggles, face mask) | Yes | No |  |
|  | Is infrared thermometer calibrated? (Look up manufacturer’s instructions on internet for calibration instructions) | Yes | No |  |
|  | Does facility perform screening on weekends? | Yes | No |  |
|  | **Isolation area** | |  | |
|  | Does the facility have a designated isolation room/ area for suspected cases of COVID 19? | Yes | No |  |
|  | Are staff trained on the use of comprehensive PPE? | Yes | No |  |
|  | Are staff trained on initial management of suspected cases? | Yes | No |  |
|  | **Notification of COVID-19 cases** |  |  |  |
|  | Is there an available phone / phone credit for calling in the notification? (Alternative would be HCW or staff call on their phones – if this the case may ask if phone credits are provided) | Yes | No |  |
|  | Is the list of phone numbers available for staff in the event of a suspect case? (Must be readily available at the screening station. Staff should have one phone number for notification to avoid needing to call multiple stakeholders) | Yes | No |  |
|  | Do facility staff know who to call if a suspect case is identified? (Ask one or two staff members) | Yes | No |  |
|  | **Water source** | |  | |
|  | Does the facility have access to clean running water? | Yes | No |  |
|  | What is the water source? | Yes | No |  |
|  | **Environmental hygiene** |  |  |  |
|  | Does the supermarket have adequate waste bins? (Adequate means having waste bins at the entrance and counter, where appropriate) | Yes | No |  |
|  | Is the environment kept tidy? | Yes | No |  |
|  | Is regular cleaning done at the supermarket? (At least twice a day) | Yes | No |  |
|  | Is there a regular cleaning schedule at the supermarket? | Yes | No |  |
|  | Are there COVID-19 posters displayed at the entrance of the supermarket? | Yes | No |  |
|  | Does the supermarket do regular disinfection of commonly touched surfaces such as the refrigerator? |  |  |  |
|  | **Toilets/Latrines** |  |  |  |
|  | Are toilet facilities available? | Yes | No |  |
|  | Is the structure in good status? | Yes | No |  |
|  | Are the toilets/Latrines clean | Yes | No |  |
|  | Do the toilet facilities offer adequate privacy? | Yes | No |  |
|  | Do the toilet facilities have anal cleansing materials? | Yes | No |  |
|  | Do the toilet facilities have hand washing facilities? | Yes | No |  |
|  | Are the hand washing facilities functional? |  |  |  |
|  | Are the hand washing facilities within a distance of 5 metres? | Yes | No |  |
|  | Is there evidence of use of functional hand washing facilities? | Yes | No |  |
|  | If yes, do the hand washing facilities have running water and soap or any disinfectant? | Yes | No |  |
|  | **Stores** |  |  |  |
|  | Is there safe storage of goods off the floor? | Yes | No |  |
|  | Is the store easily accessible to the supermarket staff? |  |  |  |
