# Supplementary material for: Extent of compliance with COVID-19 prevention and control guidelines among supermarkets in Kampala Capital City and Mukono Municipality, Uganda
Source: PLoS One. 2021 Oct 28;16(10):e0258840. doi: 10.1371/journal.pone.0258840 (PMC8553093; doi:10.1371/journal.pone.0258840)
Supplement: Attachment — Submitted filename: Point by Point response to comments.docx [file pone.0258840.s003.docx]

Makerere University School of Public Health

P.O BOX 7072, Kampala.

24^th^ July 2021.

The editor,

Plos One

**Re: Point – by – point response to the issues raised.**

Thank you for the editorial/reviewers comments raised. The response to the points raised is as below:

|  | **Point** | **Response** |
| --- | --- | --- |
| **Editor’s comments** | | |
| **1.** | Please review your reference list to ensure that it is complete and correct. If you have cited papers that have been retracted, please include the rationale for doing so in the manuscript text, or remove these references and replace them with relevant current references. Any changes to the reference list should be mentioned in the rebuttal letter that accompanies your revised manuscript. If you need to cite a retracted article, indicate the article’s retracted status in the References list and also include a citation and full reference for the retraction notice. | We have reviewed the reference list and all references are correct and complete. No retracted papers have been cited. |
| **2.** | Please include additional information regarding the data collection tool used in the study and ensure that you have provided sufficient details that others could replicate the analyses. For instance, if you developed a tool as part of this study and it is not under a copyright more restrictive than CC-BY, please include a copy, in both the original language and English, as Supporting Information. | Thank you. As part of the supporting information, we included a copy of the data collection (English version) tool that was used. We had the data collection tool in one language (English) since data was collected through observations. The data collection tool was provided in S1 Appendix, see page 7 line 25. |
| **Reviewer 1’s comments** | | |
| 1. | Please update the latest number of cases. | Thank you for this comment. The latest number of COVID-19 cases was updated as can be seen on page 4 lines 8-9 and 25 -26. |
| 2. | Would you mind providing some information on Uganda’s COVID-19 history and how the government has responded to this pandemic in the introduction section? | Thank you. On page 4, lines 9-26, we provided Uganda’s COVID-19 history and how the government has responded to this pandemic. |
| **3.** | Despite the existence of these guidelines and the public health importance of specific preventive measures, there is limited evidence on the extent of compliance with the COVID-19 prevention 14 guidelines in supermarkets. Does this limited evidence mean no paper at all? Would you please add a summary of documents complying with COVID-19 guidelines and address your research objective? | Thank you for this comment. On page 5 line 30 and page 6 lines 1-5, we have added a summary of literature related to the compliance with COVID-19 guidelines in Uganda, and addressed the research gap prior to introducing the objective. |
| **4.** | How many research assistants were recruited for the observations? | We included the number of research assistants on page 7 line 26. |
| **5.** | I feel that based on the results and discussion, the conclusion seems a little weak. Could the authors add some solid recommendations and suggestions to the public as well the government to act? | Thank you. On page 20, lines 11-15, the conclusion was strengthened. Besides, on lines 16-24, the recommendations were strengthened. |
